# Association between frailty and short- and long-term mortality in patients with critical acute myocardial infarction: Results from MIMIC-IV

**DOI:** 10.3389/fcvm.2022.1056037

**Published:** 2022-12-15

**Authors:** Weimin Bai, Benchuan Hao, Wenwen Meng, Ji Qin, Weihao Xu, Lijie Qin

**Affiliations:** ^1^Department of Emergency, Henan Provincial People’s Hospital, People’s Hospital of Zhengzhou University, People’s Hospital of Henan University, Zhengzhou, China; ^2^Medical School of Chinese People’s Liberation Army (PLA), Beijing, China; ^3^Department of Cardiology, The Second Medical Center and National Clinical Research Center for Geriatric Diseases, Chinese PLA General Hospital, Beijing, China; ^4^The Northern District of PLA General Hospital, Beijing, China; ^5^Haikou Cadre’s Sanitarium of Hainan Military Region, Haikou, China

**Keywords:** frailty, Hospital Frailty Risk Score, acute myocardial infarction, critically ill patients, mortality

## Abstract

**Background:**

Frailty has been recognized as an important prognostic indicator in patients with acute myocardial infarction (AMI). However, no study has focused on critical AMI patients. We aimed to determine the impact of frailty on short- and long-term mortality risk in critical AMI patients.

**Methods:**

Data from the Medical Information Mart for Intensive Care (MIMIC)-IV database was used. Frailty was assessed using the Hospital Frailty Risk Score (HFRS). Outcomes were in-hospital mortality and 1-year mortality. Logistic regression and Cox proportional-hazards models were used to investigate the association between frailty and outcomes.

**Results:**

Among 5,003 critical AMI patients, 2,176 were non-frail (43.5%), 2,355 were pre-frail (47.1%), and 472 were frail (9.4%). The in-hospital mortality rate was 13.8%, and the 1-year mortality rate was 29.5%. In our multivariable model, frailty was significantly associated with in-hospital mortality [odds ratio (OR) = 1.30, 95% confidence interval (CI): 1.20–1.41] and 1-year mortality [hazard ratio (HR) = 1.29, 95% CI: 1.24–1.35] as a continuous variable (per five-score increase). When assessed as categorical variables, pre-frailty and frailty were both associated with in-hospital mortality (OR = 2.80, 95% CI: 2.19–3.59 and OR = 2.69, 95% CI: 1.93–3.73, respectively) and 1-year mortality (HR = 2.32, 95% CI: 2.00–2.69 and HR = 2.81, 95% CI: 2.33–3.39, respectively) after adjustment for confounders. Subgroup analysis showed that frailty was only associated with in-hospital mortality in critically ill patients with non-ST-segment elevation myocardial infarction (STEMI) but not STEMI (*p* for interaction = 0.012). In addition, frailty was associated with 1-year mortality in both STEMI and non-STEMI patients (*p* for interaction = 0.447). The addition of frailty produced the incremental value over the initial model generated by baseline characteristics for both in-hospital and 1-year mortality.

**Conclusion:**

Frailty, as assessed by the HFRS, was associated with both in-hospital and 1-year mortality in critical AMI patients. Frailty improves the prediction of short- and long-term mortality in critical AMI patients and may have potential clinical applications.

## 1 Introduction

Frailty is a syndrome that is characterized by declined reserves of multiple organ systems in the body, leading to vulnerability to failure in the face of external stimuli, which, in turn, results in a higher risk of multiple adverse health outcomes, including falls, hospitalization, and mortality (1). Frailty is common among community-dwelling older adults, with the estimated global prevalence being 12% according to a recent systematic review (2). People with cardiovascular diseases (CVD) have an even higher prevalence of frailty. Previous meta analyses have shown that the prevalence of frailty is approximately 17.9% in CVD patients and nearly 30% in patients with acute coronary syndrome (ACS) (3, 4).

Frailty has been found to be associated with poor prognosis in several CVD. Goyal et al. found that frailty was associated with higher risk of mortality and rehospitalization in patients with heart failure with preserved ejection fraction (5). Burton et al. performed a meta-analysis and showed that pre-stroke frailty was associated with increased risk of longer-term mortality and disability in patients with acute stroke (6). Wilkinson et al.’s study indicated that there was an increase in stroke and bleeding risk in patients with atrial fibrillation with increasing frailty (7). There is also a substantial body of evidence to indicate that frailty is a prognostic indicator in patients with acute myocardial infarction (AMI) (8–10), which highlights the importance of frailty assessment in the clinical management of AMI. At present, there are more than 20 types of frailty assessment tools used in epidemiological research and clinical practice (11). Xu et al. conducted a meta-analysis and found that several frailty tools, including fried phenotype, the frailty index (FI), the FRAIL scale, the Edmonton frail scale (EFS), the Green score, and the clinical frailty scale (CFS), have been used in patients with ACS (4). Recently, Gilbert et al. developed and validated the Hospital Frailty Risk Score (HFRS), which was based on the International Statistical Classification of Diseases and Related Health Problems, Tenth Revision (ICD-10) diagnostic codes (12). The HFRS can be easily implemented in hospital electronic information systems, eliminating the inter-operator variability and assessment burden of commonly used frailty tools. The prognostic predictive role of the HFRS has been studied in a variety of patients, including patients with CVD (13), carcinoma (14), coronavirus pneumonia (15), and chronic obstructive pulmonary disease (16), as well as those receiving various surgical procedures (17–20). However, few studies have investigated the application of the HFRS in patients with AMI. Furthermore, to our knowledge, no previous study has focused on critical AMI patients.

In the present study, we analyzed data from the Medical Information Mart for Intensive Care (MIMIC)-IV database to examine the association between frailty (as assessed by the HFRS) and mortality among critical AMI patients. In addition, we investigated the incremental value of adding the HFRS to a base model that includes traditional risk factors for predicting the mortality risk of critical AMI patients.

## 2 Materials and methods

### 2.1 Data sources and study population

Data was extracted from MIMIC-IV (certification number: 10713670). This publicly available relational database is provided by the Laboratory for Computational Physiology at the Massachusetts Institute of Technology (MIT, Cambridge, MA, USA), and it includes information on critical care patients who were admitted to the intensive care unit (ICU) at the Beth Israel Deaconess Medical Center (BIDMC, Boston, MA, USA) during the period of 2008–2019. We conducted this study in compliance with the Reporting of Studies Conducted using Observational Routinely Collected Health Data (RECORD) statement.

Patients in the MIMIC-IV database who were diagnosed with AMI were eligible for the current study. The exclusion criteria were as follows: (1) patients aged < 18 years; (2) patients with a survival time < 24 h (3) organ donors; (4) patients who were pregnant, had recently given birth, or had puerperal illness; and (5) patients with missing key variables (demographic data, sequential organ failure assessment (SOFA) scores, the Charlson comorbidity index (CCI), items to construct the HFRS, and vital status at hospital discharge) for analyses. If AMI patients had more than one ICU admission record, only the first ICU admission record was included for analysis.

### 2.2 Data collection

The MIMIC-IV database includes comprehensive and high-quality data of critically ill patients admitted to the ICU at the Beth Israel Deaconess Medical Center between 2008 and 2019. ICD-9 and -10 codes were documented for specific disease diagnosis in the MIMIC-IV database. We used the following ICD codes to define and extract AMI patients: ICD-9: 410.0 (410.00, 410.01, and 410.02), 410.1 (410.10, 410.11, and 410.12), 410.2 (410.20, 410.21, and 410.22), 410.3 (410.30, 410.31, and 410.32), 410.4 (410.40, 410.41, and 410.42), 410.5 (410.50, 410.51, and 410.52), 410.6 (410.60, 410.61, and 410.62), 410.7 (410.70, 410.71, and 410.72), 410.8 (410.80, 410.81, and 410.82), and 410.9 (410.90, 410.91, and 410.92), and ICD-10: I21.0 (I21.01, I21.02, and I21.09), I21.1 (I21.11 and I21.19), I21.2 (I21.21 and I21.29), I21.3, and I21.4.

The covariates used in this study included age, sex, ethnicity (White, Black, and other), maximum SOFA scores within 24 h after ICU admission, AMI type [ST-segment elevation myocardial infarction (STEMI) and non-STEMI], and the CCI.

### 2.3 Assessment of frailty

We used the HFRS to assess the frailty status of the included critical AMI patients. The HFRS is a newly developed and validated frailty assessment tool based on ICD-10 diagnostic codes that identifies patients who are at risk of adverse healthcare outcomes. Based on the methods reported for determining the HFRS by Gilbert et al. specific weights were applied to a list of 109 ICD-10 codes, and the HFRS was calculated as the sum of the 109 ICD codes. For each patient, we calculated the HFRS based on 1 or more of 109 ICD-10-CM diagnosis codes that were coded as present on admission during the index hospitalization. We divided the patients into three groups as per their HFRS: non-frail (HFRS < 5), pre-frail (5 ≤ HFRS < 15), and frail (HFRS ≥ 15). Detailed methods and the construction of the HFRS are presented in Supplementary Table 1.

### 2.4 Outcomes

Outcomes of interest of this study were in-hospital mortality and 1-year mortality (all-cause mortality within 1 year after the date of ICU admission).

### 2.5 Statistical analysis

Baseline characteristics of the study population were presented and compared according to their different frailty statuses (non-frail, pre-frail, and frail). We reported continuous variables as the means with standard deviations (SDs) and categorical variables as frequencies and percentages. We used one-way analysis of variance to compare the differences in continuous variables and an χ2 test to compare differences in categorical variables between groups. We applied logistics regression to examine the association between frailty (as a continuous or categorical variable) and in-hospital mortality. We used a Cox regression model to investigate the association between frailty (as a continuous or categorical variable) and 1-year mortality. Age, sex, ethnicity, the CCI, AMI type, and the SOFA scores were adjusted in both the multivariable logistic and Cox regression model. The model’s predictive performance was assessed using metrics of discrimination (Harrel’s C statistic). The ΔC-statistic, integrated discrimination improvement (IDI), and net reclassification improvement (NRI) were calculated to determine the incremental predictive value of adding frailty (as a continuous variable) to the base model for in-hospital and 1-year mortality. We further conducted several subgroup analyses to assess the robustness of the results. Interactions between frailty (with the HFRS as a continuous variable) and subgroups were also tested. Finally, restricted cubic spline regression models with 5 knots were used to display the association between HFRS and in-hospital and 1-year mortality, adjusted for age, sex, ethnicity, the CCI, AMI type, and the SOFA scores. We selected the HFRS of 5 as the reference population for restricted cubic spline plots. Statistical analyses were conducted using SPSS 26.0 for Windows (SPSS Inc., Chicago, IL) and R (version 4.1.2). A *P* < 0.05 was considered statistically significant.

## 3 Results

### 3.1 Characteristics of study population

**FIGURE 1:**
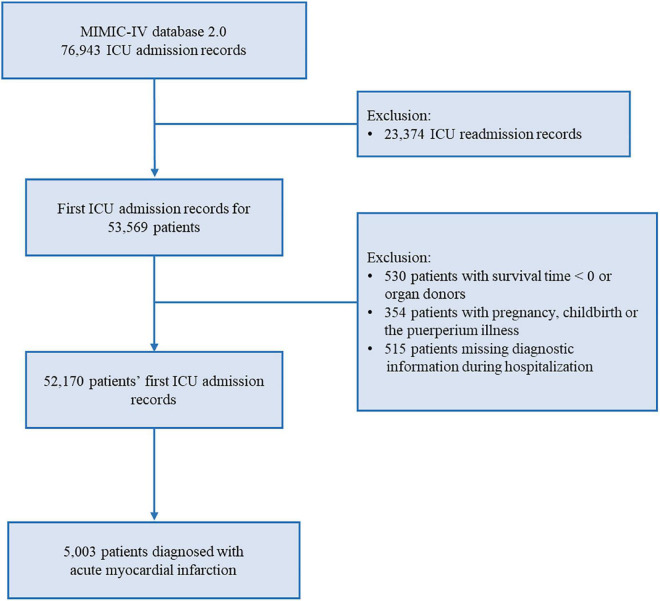
Flowchart of the detailed participants selection process.

**FIGURE 2:**
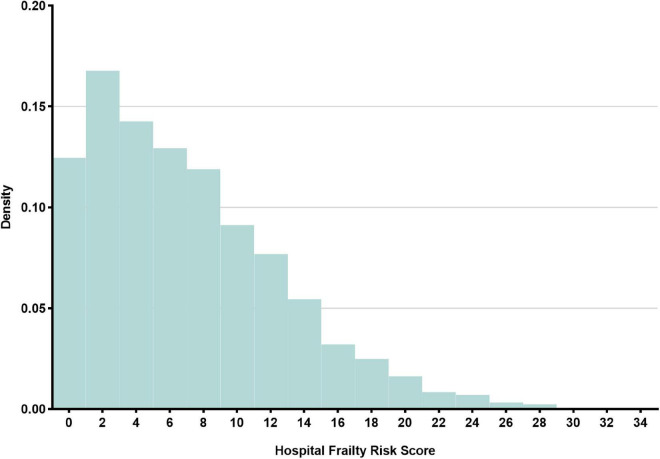
Histograms showing the distribution of the Hospital Frailty Risk Score (HFRS).

The final study population consisted of 5,003 critical AMI patients. The details of the study selection process are illustrated in Figure 1. The baseline characteristics of the participants are described in Table 1. The mean (SD) age was 69.8 (13.1) years. Males accounted for 63.3% of the participants, and the mean (SD) HFRS was 7.0 (5.7) points. The HFRS distribution is shown in Figure 2. Of the included patients, 2,176 were non-frail (43.5%), 2,355 were pre-frail (47.1%), and 472 were frail (9.4%). As expected, frail patients were older, and had higher SOFA and CCI scores, and a higher proportion of them were female and had chronic disease histories.

**TABLE 1:** Characteristics of included patients according to frailty status (*n* = 5,003).

Variable	Non-frail (n = 2176)	Pre-frail (n = 2355)	Frail (n = 472)	*p*-value
Age (years)	66.9 ± 12.9	71.6 ± 12.8	74.4 ± 12.7	<0.001
Males (*n*, %)	1,515 (69.6%)	1,399 (59.4%)	255 (54.0%)	<0.001
**Ethnicity (*n*, %)**				
White	1,377 (63.3%)	1,551 (65.9%)	296 (62.7%)	<0.001
Black	117 (5.4%)	185 (7.9%)	45 (9.5%)	
Other	682 (31.3%)	619 (26.3%)	131 (27.8%)	
SOFA scores (points)	3.5 ± 2.8	5.8 ± 3.5	6.3 ± 3.4	<0.001
**Medical history (*n*, %)**				
Cerebrovascular disease	114 (5.2%)	401 (17.0%)	175 (37.1%)	<0.001
Congestive heart failure	815 (37.5%)	1,424 (60.5%)	305 (64.5%)	<0.001
Chronic pulmonary disease	425 (19.5%)	687 (29.2%)	136 (28.8%)	
Dementia	16 (0.7%)	104 (4.4%)	99 (21.0%)	<0.001
Renal disease	279 (12.8%)	950 (40.3%)	234 (49.6%)	
Diabetes	756 (35.2%)	1,074 (45.6%)	216 (45.8%)	<0.001
Malignant cancer	98 (4.5%)	226 (9.6%)	53 (11.2%)	<0.001
STEMI	870 (40.0%)	790 (33.5%)	181 (38.3%)	<0.001
CCI (points)	5.8 ± 2.2	7.9 ± 2.5	9.0 ± 2.7	<0.001
HFRS (points)	2.1 ± 1.5	9.1 ± 2.8	19.0 ± 3.4	<0.001

SOFA, sequential organ failure assessment; STEMI, ST-segment elevation myocardial infarction; CCI, Charlson comorbidity index; HFRS, hospital frailty risk score.

### 3.2 Association between frailty and in-hospital and 1-year mortality

**FIGURE 3:**
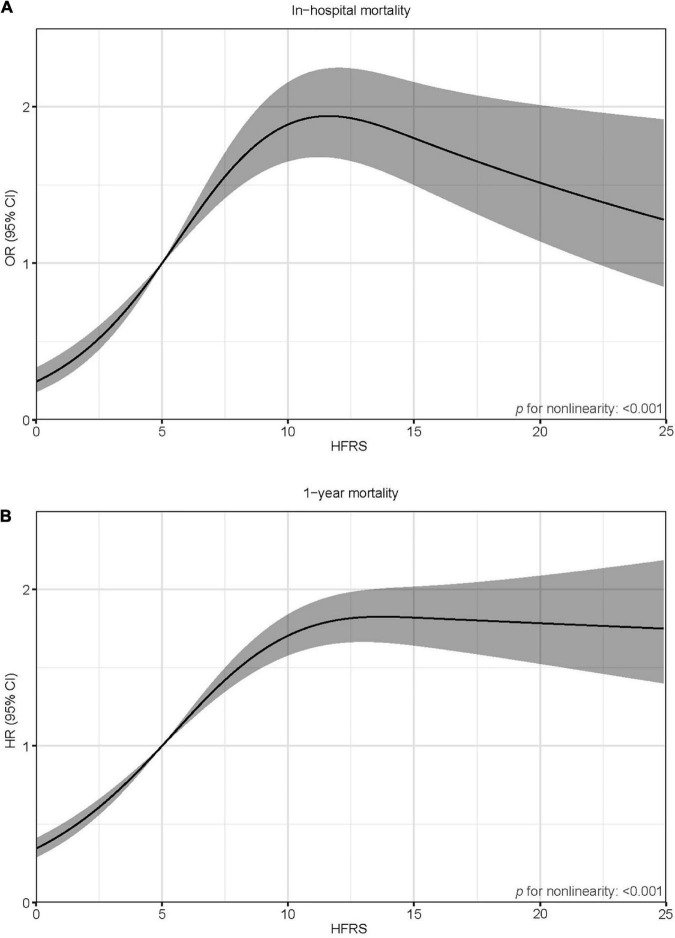
Spline curves showing the association of the HFRS as a continuous variable with in-hospital mortality **(A)** and 1-year mortality **(B)**. Spline curves were adjusted for age, sex, ethnicity, the CCI, AMI type, and SOFA score.

Six hundred and ninety patients died in the hospital (13.8%). All baseline characteristics were associated with in-hospital mortality (Supplementary Table 2). Age, sex, ethnicity (White), AMI type, CCI, SOFA, and HFRS emerged as independent predictors of in-hospital mortality (Supplementary Table 2). The HFRS was significantly associated with in-hospital mortality both as a continuous variable [per five-score increase: odds ratio (OR) = 1.30, 95% confidential interval (CI) 1.20–1.40; Figure 3] and as a categorical variable (pre-frailty: OR = 2.80, 95% CI 2.19–3.59; frailty: OR = 2.69, 95% CI 1.93–3.73; Table 2) in a multivariable model. The base model demonstrated a good discrimination ability [area under the curve (AUC) = 0.787]. The addition of the HFRS to the base model improved its ability to identify those who died in the hospital (ΔC-statistic 0.012, *p* < 0.001; Table 3). When the HFRS was included, the discrimination was slightly improved, with an IDI of 0.004 (95% CI: 0.001–0.009), and the net improvement in predicted probabilities increased significantly (NRI = 0.332; Table 3).

**TABLE 2:** Associations between frailty and in-hospital and 1-year mortality.

	Unadjusted model	Model 1*	Model 2^†^
**In-hospital mortality**	**Odds ratio (95% confidence interval)**		
HFRS (per 5-score)	1.68 (1.58–1.80)	1.62 (1.51–1.74)	1.30 (1.22–1.41)
Non-frail	Ref.	Ref.	Ref.
Pre-frail	5.55 (4.42–6.97)	5.12 (4.07–6.44)	2.80 (2.19–3.59)
Frail	6.90 (5.14–9.26)	6.07 (4.50–8.18)	2.69 (1.93–3.73)
**1-year mortality**	**Hazard ratio (95% confidence interval)**		
HFRS (per 5-score)	1.62 (1.56–1.68)	1.52 (1.47–1.58)	1.29 (1.24–1.35)
Non-frail	Ref.	Ref.	Ref.
Pre-frail	4.13 (3.60–4.75)	3.56 (3.09–4.09)	2.32 (2.00–2.69)
Frail	6.72 (5.67–7.98)	5.45 (4.58–6.48)	2.81 (2.33–3.39)

*Model 1 adjusted for age and sex. ^†^Model 2 adjusted for age, sex, ethnicity, CCI, SOFA, and AMI type (STEMI vs. Non-STEMI). HFRS, hospital frailty risk score; CCI, Charlson comorbidity index; SOFA, sequential organ failure assessment.

**TABLE 3:** Incremental value of HFRS for in-hospital and 1-year mortality.

	ΔC-statistic	*p-*value	IDI (95% CI)	NRI (95% CI)
LIn-hospital mortality				
Base model + HFRS vs. base model	0.012	<0.001	0.004 (0.001–0.008)	0.332 (0.253–0.412)
**1-year mortality**				
Base model + HFRS vs. base model	0.014	<0.001	0.020 (0.010–0.028)	0.358 (0.296–0.433)

Base model adjusted for age, sex, ethnicity, CCI, SOFA, and AMI type (STEMI vs. non-STEMI). HFRS, hospital frailty risk score; IDI, integrated discrimination improvement; NRI, net reclassification index; CI, confidence interval; CCI, Charlson comorbidity index; SOFA, sequential organ failure assessment; AMI, acute myocardial infarction; STEMI, ST-segment elevation myocardial infarction.

A total of 1,474 patients died within 1 year (29.5%). Age, sex, AMI type, the CCI, the SOFA, and the HFRS were independently associated with 1-year mortality (Supplementary Table 2). After adjustment for multiple confounders, patients with a higher HFRS had increased risk of 1-year mortality (per five-score increase: hazard ratio (HR) = 1.29, 95% CI 1.24–1.35; Figure 3); pre-frail patients and frail patients had significantly higher 1-year mortality risks compared to non-frail ones (HR = 2.32, 95% CI 2.00–2.69 and HR = 2.81, 95% CI 2.33–3.93, respectively; Table 2). The base model demonstrated a good discrimination ability (AUC = 0.741). The addition of the HFRS improved the base model’s ability to discriminate patients with 1-year mortality (ΔC-statistic 0.014, *p* < 0.001; Table 3). The resulting IDI was 0.020 (95% CI 0.010–0.028). The inclusion of the HFRS also improved the reclassification of 1-year mortality (NRI 0.358, 95% CI 0.296–0.433).

### 3.3 Subgroup analysis

In the subgroup analysis for the association between frailty and in-hospital mortality, there was significant interaction between frailty and the SOFA score and AMI type. In the SOFA subgroup, the association between frailty and in-hospital mortality in patients with an SOFA > 2 points was significantly stronger than the association in those with a SOFA ≤ 2 points (*p* for interaction < 0.001). In the AMI type subgroup, non-STEMI patients with a higher HFRS had a higher risk of in-hospital mortality (OR = 1.44, 95% CI 1.30–1.59); however, the HFRS was not associated with in-hospital mortality in critical STEMI patients (*p* for interaction = 0.012) (Table 4). In the subgroup analysis for the association between frailty and 1-year mortality, there was a significant interaction between frailty and the SOFA. The association between frailty and in-hospital mortality in patients with a SOFA > 2 points was significantly stronger than the association in those with a SOFA ≤ 2 points (*p* for interaction = 0.002) (Table 4). There was no interaction between frailty and the age subgroup for in-hospital and 1-year mortality (both *p* for interaction > 0.05).

**TABLE 4:** Subgroup analyses of the associations between frailty and in-hospital and 1-year mortality.

In-hospital mortality	Odds ratio* (95% confidence interval)		*p* for interaction
Age	≥65	<65	0.150
HFRS (per 5-score)	1.24 (1.13–1.36)	1.42 (1.19–1.70)	
SOFA	>2	≤2	<0.001
HFRS (per 5-score)	1.24 (1.135–1.35)	1.69 (1.36–2.09)	
AMI type	STEMI	Non-STEMI	0.012
HFRS (per 5-score)	1.07 (0.93–1.23)	1.44 (1.30–1.59)	
**1-year mortality**	Hazard ratio* (95% confidence interval)		***p* for interaction**
Age	≥65	<65	0.060
HFRS (per 5-score)	1.26 (1.20–1.32)	1.32 (1.19–1.48)	
SOFA	>2	≤2	0.002
HFRS (per 5-score)	1.26 (1.20–1.33)	1.43 (1.29–1.58)	
AMI type	STEMI	Non-STEMI	0.447
HFRS (per 5-score)	1.21 (1.12–1.30)	1.33 (1.26–1.41)	

*Adjusted for age, sex, ethnicity, CCI, SOFA, and AMI type (STEMI vs. non-STEMI); HFRS, hospital frailty risk score; CCI, Charlson comorbidity index, SOFA, sequential organ failure assessment; AMI, acute myocardial infarction; STEMI, ST-segment elevation myocardial infarction.

## 4 Discussion

To our knowledge, this is the first study to examine the harmful effect of frailty, as assessed by the HFRS, on short- and long-term mortality in critical AMI patients. Our main findings can be summarized as follows: (i) the HFRS to measure frailty predicts short- and long-term mortality in critical AMI patients; (ii) frailty, as assessed by the HFRS, had incremental value for mortality when added to a model containing traditional prognostic variables; and (iii) the association between frailty and short-term mortality differed across patients with different disease severities (assessed by the SOFA score) and AMI types (STEMI vs. non-STEMI), whereas the association between frailty and long-term mortality differed only across patients with different disease severities.

In the past decade, a number of studies have dealt with frailty in ACS patients and confirmed its important prognostic role (4, 8–10). A recent meta-analysis by Putthapiban et al. involving 143,301 subjects showed that frailty was strongly and independently associated with bleeding, early and late mortality in elderly with AMI (21). Despite consensus about the need for improving risk stratification in ACS patients, frailty assessment has not yet become routine clinical practice, for several reasons. First, more than 20 frailty tools have been developed. Choosing the most appropriate tool under different clinical settings is thus often a challenge for clinicians. For example, the proper frailty measure for mild ACS patients in wards and critical ACS patients in an ICU setting may not be the same. Second, the optimal timing to evaluate frailty status of patients is debatable in an acute scenario. Third, current frailty measures are mostly manual scoring systems, which places a heavy burden on clinicians.

The utilization of the HFRS could largely resolve the above barriers of routine frailty assessment in clinical practice. The HFRS can be easily integrated into electronic medical records and can be calculated immediately after the disease diagnosis process is completed. Importantly, the HFRS has been both internally and externally validated using administrative data from different patient populations and countries. Two previous studies have explored the prognostic role of the HFRS in AMI patients. Kundi et al. performed a nationwide cohort study of 785,127 Medicare fee-for-service beneficiaries aged 65 years and older in the United States (22). Of the 166,200 AMI patients included, the HFRS was associated with 30-day post-admission mortality, 30-day post-discharge mortality, and 30-day readmission, both as a continuous and categorical variable (22). In addition, Kundi et al.’s study showed that the addition of the HFRS to traditional comorbidity-based risk-prediction models significantly improved discrimination while predicting the aforementioned three outcomes (22). Kundi et al. also validated the HFRS in Turkish AMI patients. The authors obtained the data of 35,096 AMI patients diagnosed between January 1, 2018, and December 31, 2018 from national digital health record systems and found that frailty was significantly associated with 1-year readmission and 1-year mortality risk (23). Our study was in line with Kundi’s studies, that HFRS was associated with not only short-term mortality risk but also long-term mortality risk in AMI patients. Moreover, our study further confirmed the prognostic role and incremental value of the HFRS in critical AMI patients. Notably, the current risk predictive models for AMI patients or critically ill patients do not include frailty as an important risk factor. Our results show that the improvement of discrimination for short- and long-term mortality as assessed by C statistic was modest in magnitude but statistically significant with the addition of the HFRS. It was also robust, as measured by IDI and NRI. Future well-designed, high-quality cohort studies with large sample sizes are needed to confirm the prognostic role of the HFRS and determine whether adding frailty to current risk models could meaningfully improve them.

The results of our subgroup analyses offer valuable insight. First, although the HFRS was initially developed and validated in patients aged 75 years or older and has been externally validated mostly in older adults, our study showed that it was also associated with mortality risk in critical AMI patients below the age of 65 years. Researchers and clinicians generally focus on frailty in older adults, but frailty can occur in adults at any age—especially in those with chronic illnesses. Furthermore, the HFRS might capture other prognostic information besides frailty. The application of the HFRS in younger patients, especially critically ill patients, thus requires further exploration. Second, we found that the HFRS was not associated with in-hospital mortality in STEMI patients, and the association between the HFRS and in-hospital mortality in relatively mild patients was weaker than in severe patients, as assessed by the SOFA. This result indicated that frailty might have a weak or even no prognostic role in sicker patients. A previous study by Zampieri et al. also reported that the association between frailty and mortality was less evident in patients admitted with higher SOFA scores (24). Future studies are needed to further investigate this issue.

The strengths of this study include its large sample size and long-term outcomes. To the best of our knowledge, this is the first study to demonstrate the prognostic role of frailty, as assessed by the HFRS, in critical AMI patients. Our study also has several limitations. First, the development and validation of the HFRS was based on ICD-10 codes. In the MIMIC-IV database, ICD-9 and -10 co-exist for disease diagnosis. We used the conversion of ICD-9 to -10 to construct the HFRS. This might lead to the slight inaccuracy of the HFRS. Second, past disease history is often incomplete or missing in ICU settings (MIMIC-IV), which may have led to underestimation of the HFRS of our study patients. Third, important prognostic scores such as the Global Registry of Acute Coronary Events and Thrombolysis in Myocardial Infarction risk scores were not included in the multiple adjusted model owing to the lack of relevant information in the MIMIC-IV database.

## 5 Conclusion

Among critical AMI patients, frailty, as measured by the HFRS was strongly associated with short- and long-term mortality. The addition of the HFRS to traditional risk factor-based risk-prediction models significantly improved the prediction of mortality risk.

## Data availability statement

Publicly available datasets were analyzed in this study. This data can be found here: https://physionet.org.

## Ethics statement

The studies involving human participants were reviewed and approved by the Institutional Review Boards (IRB) of the Massachusetts Institute of Technology. The patients/participants provided their written informed consent to participate in this study.

## Author contributions

WX and LQ designed, supervised the study, and critically revised the manuscript. WB and JQ curated, harmonized the data, and critically reviewed all statistical methods, procedures, and results. WB, BH, and WM performed statistical analyses and drafted the manuscript. All authors listed have made a substantial, direct, and intellectual contribution to the work, and approved it for publication.
