# Supplementary material for: Association between frailty and short- and long-term mortality in patients with critical acute myocardial infarction: Results from MIMIC-IV
Source: Front Cardiovasc Med. 2022 Dec 15;9:1056037. doi: 10.3389/fcvm.2022.1056037 (PMC9797732; doi:10.3389/fcvm.2022.1056037)
Supplement: Supplementary file 1 [file Table_1.DOCX]

**Association between Frailty and Short- and Long-term Mortality in Patients with Critical Acute Myocardial Infarction: Results From MIMIC-IV**

Weimin Bai^1*^, Benchuan Hao^2,3*^, Wenwen Meng^4*^, Ji Qin^2,3^, Weihao Xu^5#^, and Lijie Qin^1#^

^1^ Department of Emergency, Henan Provincial People’s Hospital, People’s Hospital of Zhengzhou University, People’s Hospital of Henan University, Zhengzhou 463599 , China

^2^ Medical School of Chinese PLA, Beijing 100853, China

^3^ The Northern District of PLA General Hospital, Beijing 100853, China

^4^ Department of Cardiology, The Second Medical Center & National Clinical Research Center for Geriatric Diseases, Chinese PLA General Hospital, Beijing 100853, China

^5^ Haikou Cadre's Sanitarium of Hainan Military Region, Haikou 570203, China

**Table S1 Weighted scores of the 109 ICD-10 codes included in HFRS and the mapping from ICD-10 to ICD-9**

| No. | Weight | ICD-10 | ICD-10 Description | ICD-9 |
| --- | --- | --- | --- | --- |
| 1 | 7.1 | F00 | Dementia in Alzheimer's disease | (3310 AND (2900 OR 29010 OR 29011 OR 29012 OR 29013 OR 29020 OR 29021 OR 2903 OR 29040 OR 29041 OR 29042 OR 29043)) OR (3310 AND (2940 OR 29410 OR 29411 OR 29420 OR 29421)) |
| 2 | 4.4 | G81 | Hemiplegia | 342* |
| 3 | 4 | G30 | Alzheimer's disease | 3310 |
| 4 | 3.7 | I69 | Sequelae of cerebrovascular disease | 438* |
| 5 | 3.6 | R29 | Other symptoms and signs involving the nervous and musculoskeletal systems (R29.6 Tendency to fall) | 7196* OR 72989 OR 7814 OR 7816 OR 7817 OR 7819* OR 7961 |
| 6 | 3.2 | N39 | Other disorders of urinary system (including urinary tract infection and urinary incontinence) | 5990 OR 59989 OR 5999 OR 6256 OR 7883* |
| 7 | 3.2 | F05 | Delirium, not induced by alcohol and other psychoactive substances | 2930 OR 2931 |
| 8 | 3.2 | W19 | Unspecified fall | E8889 |
| 9 | 3.2 | S00 | Superficial injury of head | 9100 OR 9101 OR 9108 OR 9109 |
| 10 | 3 | R31 | Unspecified haematuria | 5997* |
| 11 | 2.9 | B96 | Other bacterial agents as the cause of diseases classified to other chapters (secondary code) | 0413 OR 0414* OR 0415 OR 0416 OR 0417 OR 0418* OR 0419 |
| 12 | 2.7 | R41 | Other symptoms and signs involving cognitive functions and awareness | 78093 OR 78097 OR 7818 OR 797 OR 7995* |
| 13 | 2.6 | R26 | Abnormalities of gait and mobility | 7197 OR 7812 |
| 14 | 2.6 | I67 | Other cerebrovascular diseases | 4359 OR 436 OR (437* NOT 4377) |
| 15 | 2.6 | R56 | Convulsions, not elsewhere classified | 7803* |
| 16 | 2.5 | R40 | Somnolence, stupor and coma | 7800* |
| 17 | 2.4 | T83 | Complications of genitourinary prosthetic device, implants and grafts | 6293* OR 9963* OR 99664 OR 99665 OR 99676 |
| 18 | 2.4 | S06 | Intracranial injury | 800* NOT (8000* OR 8005*) OR 801* NOT (8010* OR 8015*) OR 803* NOT (8030* OR 8035*) OR 804* NOT (8040* OR 8045*) OR 85* OR 9070 |
| 19 | 2.3 | S42 | Fracture of shoulder and upper arm | 810* OR 811* OR 812* |
| 20 | 2.3 | E87 | Other disorders of fluid, electrolyte and acid-base balance | 276* NOT 2765* |
| 21 | 2.3 | M25 | Other joint disorders, not elsewhere classified | 7190* OR 7191* OR (7194* NOT 71944) OR 7195* OR 7198* OR 7199* |
| 22 | 2.3 | E86 | Volume depletion | 2765* |
| 23 | 2.2 | R54 | Senility | 797 |
| 24 | 2.1 | Z50 | Care involving use of rehabilitation procedures | V57* |
| 25 | 2.1 | F03 | Unspecified dementia | ((290* NOT 2904*) OR 2942*) NOT 3310 |
| 26 | 2.1 | W18 | Other fall on same level | E8846 OR E8859 OR E8888 OR E9177 OR E9178 |
| 27 | 2 | Z75 | Problems related to medical facilities and other health care | V605 OR V63* |
| 28 | 2 | F01 | Vascular dementia | 2904* |
| 29 | 2 | S80 | Superficial injury of lower leg | 916* OR 9241* |
| 30 | 2 | L03 | Cellulitis | 681* OR 682* |
| 31 | 1.9 | H54 | Blindness and low vision | 369* |
| 32 | 1.9 | E53 | Deficiency of other B group vitamins | 266* |
| 33 | 1.8 | Z60 | Problems related to social environment | V603 OR V624 |
| 34 | 1.8 | G20 | Parkinson's disease | 3320 |
| 35 | 1.8 | R55 | Syncope and collapse | 7802 |
| 36 | 1.8 | S22 | Fracture of rib(s), sternum and thoracic spine | 8052 OR 8053 OR 8062* OR 8063* OR (807* NOT (8075 OR 8076)) OR 809* |
| 37 | 1.8 | K59 | Other functional intestinal disorders | 5640* OR 5645 OR 5646 OR 5647 OR 5648* OR 5649 |
| 38 | 1.8 | N17 | Acute renal failure | 5836 OR 5837 OR 584* |
| 39 | 1.7 | L89 | Decubitus ulcer | 7070* OR 7072* |
| 40 | 1.7 | Z22 | Carrier of infectious disease | V02* |
| 41 | 1.7 | B95 | Streptococcus and staphylococcus as the cause of diseases classified to other chapters | 0410* OR 0411* OR 0412 |
| 42 | 1.6 | L97 | Ulcer of lower limb, not elsewhere classified | 7071* |
| 43 | 1.6 | R44 | Other symptoms and signs involving general sensations and perceptions | 7801 |
| 44 | 1.6 | K26 | Duodenal ulcer | 532* |
| 45 | 1.6 | I95 | Hypotension | 458* |
| 46 | 1.6 | N19 | Unspecified renal failure | 586 |
| 47 | 1.6 | A41 | Other septicaemia | 0381* OR 0383 OR 0384* OR 0388 OR 0389 OR 99591 |
| 48 | 1.5 | Z87 | Personal history of other disease and conditions | 30250 OR V126* OR V1270 OR V1271 OR V1279 OR V13* OR V155* OR V1582 OR V219 OR V470 OR V499 |
| 49 | 1.5 | J96 | Respiratory failure, not elsewhere classified | 51851 OR 51853 OR 51881 OR 51883 OR 51884 |
| 50 | 1.5 | X59 | Exposure to unspecific factor | E9288 OR E9289 |
| 51 | 1.5 | M19 | Other arthrosis | (7151* NOT (71515 OR 71516)) OR (7152* NOT (71525 OR 71526)) OR (7153* NOT (71534 OR 71535 OR 71536)) OR 71590 OR 71591 OR 71592 OR 71593 OR 71597 OR 71598 |
| 52 | 1.4 | G40 | Epilepsy | 345* |
| 53 | 1.4 | M81 | Osteoporosis without pathological fracture | 7330* |
| 54 | 1.4 | S72 | Fracture of femur | 820* OR 821* |
| 55 | 1.4 | S32 | Fracture of lumbar spine and pelvis | 8054 OR 8055 OR 8056 OR 8057 OR 8066* OR 808* |
| 56 | 1.4 | E16 | Other disorders of pancreatic internal secretion | 251* NOT (2510 OR 2513) |
| 57 | 1.4 | R94 | Abnormal results of function studies | 794* NOT 79401 |
| 58 | 1.4 | N18 | Chronic renal failure | 585* |
| 59 | 1.3 | R33 | Retention of urine | 78820 OR 78829 |
| 60 | 1.3 | R69 | Unknown and unspecified causes of morbidity | 79989 OR 7999 OR V419 |
| 61 | 1.3 | N28 | Other disorders of kidney and ureters, not elsewhere classified | 5903 OR 5930 OR 5931 OR 5932 OR 5938* OR 5939 |
| 62 | 1.2 | R32 | Unspecified urinary incontinence | 78830 |
| 63 | 1.2 | G31 | Other degenerative disease of the nervous system, not elsewhere classified | 3308 OR 3309 OR 33111 OR 33119 OR 3312 OR 3316 OR 3317 OR 33182 OR 33183 OR 33189 OR 3319 |
| 64 | 1.2 | Y95 | Nosocomial condition | 1369 |
| 65 | 1.2 | S09 | Other and unspecified injuries of head | 8726* OR 8727* OR 8728 OR 8729 OR 87340 OR 87350 OR 8738 OR 95901 |
| 66 | 1.2 | R45 | Symptoms and signs involving emotional state | 3079 OR 3089 OR 78095 OR 7992* OR V6284 OR V6285 |
| 67 | 1.2 | G45 | Transient cerebral ischaemic attacks and related syndromes | 435* OR 4377 |
| 68 | 1.1 | Z74 | Problems related to care-provider dependency | V4984 OR V604 |
| 69 | 1.1 | M79 | Other soft tissue disorder, not elsewhere classified | 71944 OR (729* NOT (7294 OR 72982 OR 72989 OR 72991)) |
| 70 | 1.1 | W06 | Fall involving bed | E8844 |
| 71 | 1.1 | S01 | Open wound of head | 8511* OR 8513* OR 8515* OR 8517* OR 8519* OR 8521* OR 8523* OR 8525* OR 8531* OR 8541* OR (870* NOT (8703 OR 8704)) OR 8720* OR 8721* OR (873* NOT (87329 OR 87339 OR 87340 OR 87350 OR 87363 OR 87373)) |
| 72 | 1.1 | A04 | Other bacterial intestinal infections | 008* NOT (0086* OR 0088) |
| 73 | 1.1 | A09 | Diarrhoea and gastroenteritis of presumed infectious origin | 0091 OR 0093 |
| 74 | 1.1 | J18 | Pneumonia, organism unspecified | 485 OR 486 |
| 75 | 1 | J69 | Pneumonitis due to solids and liquids | 507* |
| 76 | 1 | R47 | Speech disturbances, not elsewhere classified | 7843 OR 7845* OR V414 |
| 77 | 1 | E55 | Vitamin D deficiency | 2680 OR 2689 |
| 78 | 1 | Z93 | Artificial opening status | V44* |
| 79 | 1 | R02 | Gangrene, not elsewhere classified | 7854 |
| 80 | 0.9 | R63 | Symptoms and signs concerning food and fluid intake | 783* NOT (7834* OR 7837) |
| 81 | 0.9 | H91 | Other hearing loss | 38801 OR 38811 OR 3882 OR 3897 OR 3898 OR 3899 |
| 82 | 0.9 | W10 | Fall on and from stairs and steps | E880* |
| 83 | 0.9 | W01 | Fall on same level from slipping, tripping and stumbling | E8880 OR E8881 |
| 84 | 0.9 | E05 | Thyrotoxicosis (hyperthyroidism) | 242* |
| 85 | 0.9 | M41 | Scoliosis | (7373* NOT 73733) OR 73743 |
| 86 | 0.8 | R13 | Dysphagia | 7872* |
| 87 | 0.8 | Z99 | Dependence on enabling machines and devices | V4511 OR (V46* NOT V4614) |
| 88 | 0.8 | U80 | Agent resistant to penicillin and related antibiotics | V090 OR V091 |
| 89 | 0.8 | M80 | Osteoporosis with pathological fracture | 7330* AND (7331* OR V1351) |
| 90 | 0.8 | K92 | Other diseases of digestive system | 538 OR 578* |
| 91 | 0.8 | I63 | Cerebral infarction | 43301 OR 43311 OR 43321 OR 43331 OR 43381 OR 43391 OR 43401 OR 43411 OR 43491 |
| 92 | 0.7 | N20 | Calculus of kidney and ureter | 592* |
| 93 | 0.7 | F10 | Mental and behavioural disorders due to use of alcohol | 291* OR 303* |
| 94 | 0.7 | Y84 | Other medical procedures as the cause of abnormal reaction to the patient | E8789 OR E879* |
| 95 | 0.7 | R00 | Abnormalities of heart beat | 7850 OR 7851 OR 4278* |
| 96 | 0.7 | J22 | Unspecified acute lower respiratory infection | 5198 |
| 97 | 0.6 | Z73 | Problems related to life-management difficulty | V4985 OR V695 |
| 98 | 0.6 | R79 | Other abnormal findings of blood chemistry | 7906 OR 79091 OR 79092 OR 79095 |
| 99 | 0.5 | Z91 | Personal history of risk factor, not elsewhere classified | V150* OR V154* OR V1559 OR V1581 OR V1588 OR V1589 OR V4031 OR V4512 |
| 100 | 0.5 | S51 | Open wound of forearm | 88100 OR 88101 OR 88110 OR 88111 OR 88120 |
| 101 | 0.5 | F32 | Depressive episode | 2962* OR 29682 OR 2980 OR 311 |
| 102 | 0.5 | M48 | Spinal stenosis (secondary code only) | 7215 OR 7216 OR 7217 OR 7218 OR 7230 OR 7240* |
| 103 | 0.4 | E83 | Disorders of mineral metabolism | 275* |
| 104 | 0.4 | M15 | Polyarthrosis | 7150* OR 7158* OR 71590 |
| 105 | 0.4 | D64 | Other anaemias | 285* NOT (2851 OR 2852*) |
| 106 | 0.4 | L08 | Other local infections of skin and subcutaneous tissue | 0390 OR 68600 OR 68609 OR 6868 OR 6869 |
| 107 | 0.3 | R11 | Nausea and vomiting | 07882 OR 5362 OR 56987 OR 7870* |
| 108 | 0.3 | K52 | Other noninfective gastroenteritis and colitis | 5357* OR 558* |
| 109 | 0.1 | R50 | Fever of unknown origin | 7806* NOT (78064 OR 78065) |

* All subcodes contained in this code.

"AND", "OR" and "NOT" follow the logic of Boolean operations.

**Table S2. Associations of baseline characteristics with in-hospital and 1-year mortality**

|  | In-hospital mortality | | 1-year mortality | |
| --- | --- | --- | --- | --- |
|  | Univariate model | Multivariate model | Univariate model | Multivariate model |
| Age (per 1 year) | 1.03 (1.02-1.04) | 1.02 (1.01-1.03) | 1.04 (1.03-1.05) | 1.03 (1.02-1.03) |
| Gender (male vs. female) | 0.75 (0.64-0.88) | 0.75 (0.63-0.91) | 0.71 (0.64-0.78) | 0.81 (0.73-0.90) |
| Ethnicity |  |  |  |  |
| White | 0.73 (0.61-0.87) | 0.75 (0.63-0.91) | 1.08 (0.89-1.31) | 0.96 (0.85-1.08) |
| Black | 0.92 (0.67-1.28) | 0.75 (0.52-1.09) | 0.93 (0.82-1.04) | 0.91 (0.73-1.12) |
| AMI type (STEMI vs. non-STEMI) | 1.25 (1.06-1.47) | 1.55 (1.29-1.86) | 1.02 (0.92-1.13) | 1.26 (1.13-1.40) |
| CCI (per score increase) | 1.20 (1.17-1.24) | 1.09 (1.05-1.13) | 1.25 (1.23-1.27) | 1.13 (1.11-1.16) |
| SOFA (per score increase) | 1.29 (1.26-1.33) | 1.28 (1.25-1.31) | 1.16 (1.14-1.17) | 1.13 (1.11-1.15) |
| HFRS (per 5 scores increase) | 1.68 (1.58-1.80) | 1.30 (1.20-1.41) | 1.62 (1.26-1.68) | 1.29 (1.24-1.35) |

AMI, acute myocardial infarction; STEMI, ST-segment elevation myocardial infarction; CCI, Charlson comorbidity index, SOFA, sequential organ failure assessment; HFRS, hospital frailty risk score.
